# Supplementary material for: Effects of taurine supplementation on metabolic health and biological aging in healthcare workers: A protocol for a triple-blinded, Bayesian-optimized phase II randomized controlled trial
Source: PLoS One. 2026 May 27;21(5):e0350389. doi: 10.1371/journal.pone.0350389 (PMC13215551; doi:10.1371/journal.pone.0350389)
Supplement: S1 File — (PDF) [file pone.0350389.s002.pdf]

Protocol contributors:

1. Mandy Hiu Man CHU,1 Email: hiumanchu@gmail.com
2. Jacky Ka Ming LAI, 1 Email: kaminglai@link.cuhk.edu.hk
3. Anna LEE, 1 Email: annalee@cuhk.edu.hk
4. William Ka Kei WU, 1 Email: wukakei@cuhk.edu.hk
5. Ziheng HUANG, 1 Email: zihenghuang@cuhk.edu.hk
6. Henry Man Kin WONG, Email: henrymkwong@cuhk.edu.hk
7. Lap Tin HO, 1 Email: hlt223@ha.org.hk
8. Hao SU, 1 Email: suhao@link.cuhk.edu.hk
9. Samantha Sze Man HO, 1 Email: samanthaho@doctors.org.uk
10. Xinbo XU, 1 Email: xinboxu@cuhk.edu.hk
11. Warren PAVEY,2 Email: w.pavey@hlri.org.au
12. David J. R. MORGAN,2,3 Email: david.morgan@health.wa.gov.au
13. Matthew Tak Vai CHAN, 1 Email: mtvchan@cuhk.edu.hk
14. Kwok Ming HO,1,# Email: kmho@cuhk.edu.hk

[1] Department of Anaesthesia & Intensive Care, the Chinese University of Hong Kong and Prince of Wales Hospital, Shatin, Hong Kong SAR, China.

[2] Institute of Heart and Lung Research Institute, Perth, Western Australia, 6150.

[3] Department of Intensive Care Medicine, Fiona Stanley Hospital, Perth, Western Australia, 6150.

a. **Title:**

Taurine **O**r **P**lacebo For Healthcare Workers: the TOP Healthcare workers' Bayesian Optimized Phase II randomized-controlled trial

b(i). **Research in context:**

(i) Taurine, a conditionally essential amino acid, is not used by the body for protein synthesis and exists in high concentrations in energy-demanding organs such as the brain, retina, heart, and skeletal muscles. Its abundance invariably reduces as animals and humans age (1). A recent multinational study found that taurine supplementation increased lifespan in middle-aged mice by 10-12% and health span (including a lower fat mass, better bone density, reductions in fasting blood sugar and mitochondrial and DNA damage) in older nonhuman primates (equivalent to 45-year-old humans) compared to control animals. Taurine improves cellular and organ function in multiple ways and has been labelled as one of the 'Longevity amino acids' (2). A meta-analysis showed that taurine supplementation could reduce glycated haemoglobin (HbA1c) level by 0.4% (3); but the validity of this result was limited by small number of patients (N=209 from five trials), short supplementation duration (<16 weeks) and, most importantly, the largest randomised-controlled-trial (RCT) (n=63) had a high-risk of bias in random-sequence generation and allocation concealment (3). Another subsequent RCT (N=120) in diabetic patients showed that taurine supplementation (1g/day x 8 weeks) resulted in lower serum insulin levels, Homeostatic-Model-Assessment-of-Insulin-Resistance, and biomarkers of endothelial dysfunction but not HbA1c, likely related to the short duration of supplementation relative to the long half-life of HbA1c (4).

(ii) Reducing healthcare burden is paramount as the Hong Kong population is rapidly ageing. Currently, many professional nutritional guidelines have largely focused on macronutrients. Recent studies have revealed the importance of nutritional elements in promoting healthy ageing (2). **An adequately-powered RCT with sufficient duration of supplementation is essential to confirm whether taurine can promote healthy ageing.** If it is proven to be effective, this trial will change international nutritional guidelines and public health policies, reduce healthcare utilisation, and improve quality of life for the community at large. Conversely, if the results are negative (despite adequate statistical power and duration of supplementation), taurine supplementation should not be used by the public, reducing society's unnecessary expenditure on an ineffectual therapy.

b(ii). **Introduction:**

Ageing population is an important issue around the world including Hong Kong. The proportion of elderly persons aged ≥65 in Hong Kong rose from 13% in 2011 to 20% in 2021; median age of the population increased from 41.7 to 46.3 years during the same period. Furthermore, an increasing number of people are frail with comorbidities affecting their quality of life and increasing burden on the healthcare system. Recent research suggested that frailty and many chronic health conditions are pathogenically linked to 'accelerated biological ageing' (5) caused by DNA-methylation, spontaneous somatic mutations such as Clonal-Haematopoiesis of Indeterminate-Potential, mitochondrial dysfunction, endoplasmic reticulum (ER) stress, and accumulation of advanced-glycation-end-products (AGEs) (6-8).

Exercise has consistently been shown to improve human health including ageing-related cognitive decline (9) and frailty (10). A combination of exercise with a nutritional intervention between 1 and 12 months has been shown to reduce biological age (11-13). However, in the secondary analysis of a factorial-designed RCT over a 12-month period, diet alone could decrease biological age more than

exercise alone (-2.4 vs -0.2 years, $p<0.05$) (13). A recent systematic review also showed that combining exercise with hypocaloric diet did not further improve glycaemic control in obese diabetic adults (14), suggesting some micronutrients may be critical in promoting healthy ageing (2).

Taurine (2-aminoethanesulfonic acid) is essential in infants because its deficiency may hinder brain development (15). Human milk contains taurine and formula milk is supplemented with taurine to compensate for insufficient endogenous taurine synthesis. In adults, the primary source of taurine is the diet, especially seafood and dark meat, with an intake seldom exceeds 400mg/day. Interestingly, blood taurine levels can be increased after exercise (1); with some authors suggested that taurine may play a part to mediate the metabolic health benefits of exercise (1,16).

The mechanisms through which taurine may improve health have been summarised in our recent review (17), and by others (18). In brief, taurine supplementation improves mitochondrial and ER function, pancreatic β–cells survival, bone, retinal and mental health in animal studies. In small human studies, taurine improves glycaemic control, exercise capacity, and myocardial function. Taurine interacts with gut microbiome, increases bile acid conjugation (19), stimulates glucagon-like-peptide-1 (GLP-1) secretion, and inhibits sodium-glucose-transporter-1 (SGLT-1) non-competitively (with 80% reductions in Vmax and Michaelis-Menten-constant for glucose) (20-24). Gut microbiome and bile acids are key mediators of cardiometabolic health (25,26) while SGLT-1 inhibition reduces glucose absorption (24) and postprandial hyperglycaemia (27). Taurine is also a reactive-carbonyl-species (RCSs) scavenger (through a competitive Schiff-base reaction) (28) and can reduce haemoglobin glycation (29) and plasma AGEs — as demonstrated in a small RCT (30).

In summary, despite the strong experimental evidence supportive of taurine's health benefits, high-quality human data does not exist. Taurine is synthesized commercially in a pure form (hence also suitable for the vegans), inexpensive (<US$1/g), and considered safe for human consumption at a dose <6g/day by the European Food Safety Authority (31). There is an urgent need to identify innovative ways to improve human health span (and not just lifespan) by minimising accelerated biological ageing (32). Before an adequately powered RCT assessing the effectiveness of taurine in improving long-term human health is conducted, a high-quality phase II trial assessing the medium-term metabolic benefits of taurine supplementation is need.

c. **Aims and Hypotheses to be Tested:**

We aim to determine whether compared to placebo, oral taurine supplementation (3g/day for 6 months) will

(a) improve glucose metabolism resulting in reduced HbA1c among adult healthcare workers,

(b) improve blood low-density-lipoprotein (LDL) and triglyceride levels, biological age — as measured by the PhenoAge model (32) — and accumulation of AGEs; and

(c).

We hypothesize that taurine supplementation will:

1. improve glucose metabolism resulting in reduction in HbA1c; and
2. reduce biological age, and LDL and triglyceride, and AGEs levels

d. **Plan of Investigation:**

(i) Study design

The proposed study is a parallel-arm design, superiority, double-blinded, stratified phase II Bayesian Optimized RCT. Our proposed RCT aims to overcome the existing studies limitation, and to confirm the mechanisms through which taurine could exert its health benefit., as described in our recent review (17).

(ii) Methods

After obtaining written informed consent, healthcare worker participants will be randomly allocated to receive either oral taurine or identical-looking capsules daily for a 6-month period with 1:1 allocation ratio using computer-generated sequence (with variable block sizes), stratified by' chronological age (≤45 vs >45-year-old) and pre-existing diabetic status. Exclusion criteria include those who: (a) are taking taurine prior to enrolment, have (b) chronic renal disease (eGFR<30ml/min: as taurine can accumulate in renal failure) (35), (c) insulin-dependent diabetes mellitus, (d) a bleeding disorder (e.g. platelet count $<100x10^9/L$ or treated with dual antiplatelet agents: as taurine has potential antiplatelet activity in vitro) (36), (e) received active chemo/immunotherapy <30 days before enrolment or likely to receive such treatment during the study, or (f) are pregnant or planning to become pregnant.

We will use taurine capsules produced by Optima Ovest, the company from the Western Australia. The placebo is a proprietary product from Colorcon called StarCap. These capsules will be stored in our research office. Participants will be given the bottles of study capsules as allocated once enrolled. REDCap database will be used for randomization, treatment allocation, and data collection.

Intervention: Identical-looking placebo or taurine (1000mg capsule x3 orally in one sitting per day) is the study intervention - a dose is higher than that from standard daily dietary intake (37), well within the safe recommended dose (31), and similar to the (equivalent) dose used in the recent nonhuman primate study (1). Significant side effects were not reported in previous human RCTs using similar doses (3,4,30). Participants will be advised to take the study capsules in the morning but taking it at other times is also allowed. At the end of study, unused study capsules will be counted to assess compliance. The participants will be allowed to continue with their normal diet and usual medications but will be advised to take the study capsules one hour apart from their usual medications, even though significant interactions between taurine and commonly used medications have not been reported.

Primary outcome: Difference in the proportion of patients who have a reduced HbA1c concentration comparing 6-month to baseline between the two treatment groups.

Secondary outcomes: Difference in the changes from baseline to 6-month follow-up between the two groups in (a) LDL and triglyceride levels, (b) PhenoAge (32), (c) body weight, (d) International Physical Activity Questionnaire (IPAQ)(**Appendix 1**) (38), (e) Center for Epidemiologic Studies Depression Scale (CES-D)(**Appendix 2**) (39), (f) systolic and diastolic blood pressure, (g) AGEs index (measured non-invasively using the skin autofluorescence technology) (40), insulin and HOMA-IR, and Brain-derived neurotrophic factor (BDNF) levels.

Tertiary outcome: microbiome sub-study - Eighty participants will be invited to participate in a

sub-study (40/group). Faecal samples at baseline and at 6-month follow-up will be obtained and their gut microbiome between the two groups, as well as between baseline and at 6-month follow-up, will be compared including the differences in gut microbiota diversity in relation to changes in plasma taurine (19), and methylglyoxal (a major precursor of AGEs) (8,30) levels.

(iii) Subjects (with justification on the sample size) Having a healthy healthcare workforce is paramount in achieving a sustainable healthcare system as noted in many countries during the COVID-19 pandemic. This RCT will be conducted primarily in healthcare workers at the New Territories East Cluster of Hospitals including Prince of Wales Hospital, North District Hospital and Alice Ho Miu Ling Nethersole Hospital. Poster will be posted in these hospitals for promotion purpose. As healthcare workers are intrinsically interested in maintaining their health, they are likely to adhere to the study protocol. All healthcare-related workers who can read and understand written English or Chinese translated version of the study questionnaires will be invited to participate, including doctors, nurses, physiotherapists, dietitians, or research-related workers. Our sample size is based on the primary outcome – difference proportion of participants with a reduced HbA1c at 6-month compared to baseline between the two groups using a parallel design with 1:1 allocation.. Taurine was previously shown to reduce HbA1c by 0.41% (95%CI 0.09-0.74; p=0.001) (3). In this BOP2 trial, conditions for the stopping for superiority or futility will be based on absence of an informative prior, HbA1c concentrations will be reduced after 6-month of study intervention in the experimental and the control groups in 40% and 10% (possibly due to Hawthorne effect), respectively. The margin of meaningful difference between the two groups is set at 10%. Assuming one-sided type I error of 10% is acceptable to prove superiority or futility, this trial will have 80% with a total sample size of 80. Three interim analyses after enrolling 20, 40 and 60 participants will be performed to facilitate the BOP2 technique (Appendix 3). .

(iv) Data processing and analysis

Data Collection: We will record participants' age, weight, height, comorbidities, usual medications, and dietary restrictions. In addition to testing for HbA1c, LDL and triglyceride concentrations, the following fasting blood tests - albumin, creatinine, C-reactive protein, lymphocyte percentage, red blood cell distribution width, mean cell volume, white blood cell count, alkaline phosphatase, glucose (to compute the PhenoAge) (32), taurine, tauroursodeoxycholic, insulin, BDNF, and methylglyoxal levels - will be obtained at baseline and 6-month. The participants will be advised to take the study capsules in the morning between 1 and 3 hours prior to the 6-month follow up blood tests. Taurine, tauroursodeoxycholic, insulin, BDNF, and methylglyoxal levels will be analysed by the ELISA assays. For the microbiome sub-study, stool samples are aliquoted into cell culture cryogenic tubes and immediately stored at -80^{0}C for metagenomic sequencing locally.

Data analysis: Continuous variables will be reported as mean (SD) or median (interquartile range). Mann-Whitney test (or t-test when appropriate), including 95% confidence interval for differences between groups, will be used to analyse the outcomes. Shotgun metagenomic sequencing and paired comparisons will be conducted when comparing baseline and 6-month follow-up microbiome genus- and species-level abundance data, and whether any changes are related to the plasma taurine and methylglyoxal levels.

A subgroup analysis by excluding participants allocated to the taurine group who have 6-month plasma taurine levels less than the mean plasma taurine level of the control group, suggestive of

non-compliance to the study intervention, will be conducted. A one-sided p-value <0.10 without Bonferroni adjustment will be taken as significant for the primary outcome in this phase II trial. Continuous outcome measures will be analyzed by analysis of covariance (ANCOVA) (while adjusting for baseline data), and a p-value <0.05 without Bonferroni adjustment will be considered significant for these secondary outcome measures. Furthermore, the associations among these continuous outcome measures will be explored to advance our understanding of the mechanisms underlying accelerated biological aging.

(v) Potential pitfalls and contingency plans

Safety and tolerability: A two-member independent data safety monitoring committee (DSMC) will be formed prior to ethics application. Previous human taurine RCT did not report significant increases in adverse events (AEs) (3,4,30). A pre-plan safety margin is set to un-blind the results to the DSMC should the relative risk of AEs is >3. If the AE rate is in favour of the control group, the trial will be ceased. The reportable AEs include (a) gastrointestinal upset requiring medical attention or cessation of the study capsules, (b) allergic skin rashes, (c) spontaneous hypoglycaemia (<4mmol/L), (d) unexpected bleeding from any sites after commencement of study capsules, and (e) new symptoms the participants believe are related to the study medications resulting in cessation of study capsules.

The placebo capsules contain high-purity, spray-dried, National Formulary standard, lactose monohydrate, a purified form of milk sugar and free of milk protein. It is an inactive substance that commonly used as a placebo filler due to its characteristics of stability, compressibility and neutral taste. It is recorded as a generally recognized as safe (GRAS) food substance in the U.S. Food and Drug Administration (41). Research shows that people with lactose intolerance are able to intake up to 12 grams of lactose with only mild symptoms or even no symptoms (42).

Funding This Phase II trial is internally funded by the Department of Anaesthesia and Intensive Care, the Prince of Wales Hospital and the Chinese University of Hong Kong.

Ethical considerations: Before obtaining written informed consent, the purpose of the study, procedures, risks and benefits of participation, and the time commitment involved will be explained to eligible patients by study research staff. Participants will also be informed that benefits from taking taurine remain scientifically unproven, and results may vary from person to person. Patients may withdraw from the study without prejudice at any time during the study. Data will be kept confidential in secure offices of the Department of Anaesthesia and Intensive Care for seven years. Only group data will be published. Approval for the project will be obtained from The Joint Chinese University of Hong Kong-New Territories East Cluster Clinical Research Ethics Committee. The study will adhere to local laws, Declaration of Helsinki, ICH-GCP and institutional policies.

Participants will be able to obtain all the blood test results including their estimated biological age, and their group allocation at the end of the study. Considering the travel expense participants need, transportation reimbursement (up to HK$100) will be issued to participants who require transportation to the study centre. No other financial incentives or compensation will be provided to the participants.

e. **Key References:**

1. Singh P, Gollapalli K, Mangiola S, Schranner D, Yusuf MA, Chamoli M, et al. Taurine deficiency as a driver of aging. Science 2023;380(6649):eabn9257. doi: 10.1126/science.abn9257
2. Ames BN. Prolonging healthy aging: Longevity vitamins and proteins. Proc Natl Acad Sci U S A 2018;115(43):10836-10844. doi: 10.1073/pnas.1809045115.
3. Tao X, Zhang Z, Yang Z, Rao B. The effects of taurine supplementation on diabetes mellitus in humans: A systematic review and meta-analysis. Food Chem (Oxf) 2022;4:100106. doi: 10.1016/j.fochms.2022.100106.
4. Moludi J, Qaisar SA, Kadhim MM, Ahmadi Y, Davari M. Protective and therapeutic effectiveness of taurine supplementation plus low calorie diet on metabolic parameters and endothelial markers in patients with diabetes mellitus: a randomized, clinical trial. Nutr Metab (Lond) 2022;19(1):49. doi: 10.1186/s12986-022-00684-2.
5. Ji L, Jazwinski SM, Kim S. Frailty and Biological Age. Ann Geriatr Med Res 2021;25(3):141-149. doi: 10.4235/agmr.21.0080.
6. Nachun D, Lu AT, Bick AG, Natarajan P, Weinstock J, Szeto MD, et al.; NHLBI Trans-Omics for Precision Medicine (TOPMed) Consortium. Clonal hematopoiesis associated with epigenetic aging and clinical outcomes. Aging Cell 2021;20(6):e13366. doi: 10.1111/acel.13366
7. Taylor RC, Hetz C. Mastering organismal aging through the endoplasmic reticulum proteostasis network. Aging Cell 2020;19(11):e13265. doi: 10.1111/acel.13265.
8. Senatus L, MacLean M, Arivazhagan L, Egaña-Gorroño L, López-Díez R, Manigrasso MB, et al. Inflammation Meets Metabolism: Roles for the Receptor for Advanced Glycation End Products Axis in Cardiovascular Disease. Immunometabolism 2021;3(3):e210024. doi: 10.20900/immunometab20210024.
9. Augusto-Oliveira M, Arrifano GP, Leal-Nazaré CG, Santos-Sacramento L, Lopes-Araújo A, Royes LFF, et al. Exercise Reshapes the Brain: Molecular, Cellular, and Structural Changes Associated with Cognitive Improvements. Mol Neurobiol 2023;60(12):6950-6974. doi: 10.1007/s12035-023-03492-8.
10. Yi M, Zhang W, Zhang X, Zhou J, Wang Z. The effectiveness of Otago exercise program in older adults with frailty or pre-frailty: A systematic review and meta-analysis. Arch Gerontol Geriatr 2023;114:105083. doi: 10.1016/j.archger.2023.105083.
11. Lohman T, Bains G, Cole S, Gharibvand L, Berk L, Lohman E. High-Intensity interval training reduces transcriptomic age: A randomized controlled trial. Aging Cell 2023;22(6):e13841. doi: 10.1111/acel.13841.
12. Fitzgerald KN, Campbell T, Makarem S, Hodges R. Potential reversal of biological age in women following an 8-week methylation-supportive diet and lifestyle program: a case series. Aging (Albany NY) 2023;15(6):1833-1839. doi: 10.18632/aging.204602.
13. Ho E, Qualls C, Villareal DT. Effect of Diet, Exercise, or Both on Biological Age and Healthy Aging in Older Adults with Obesity: Secondary Analysis of a Randomized Controlled Trial. J Nutr Health Aging 2022;26(6):552-557. doi: 10.1007/s12603-022-1812-x.
14. Memelink RG, Hummel M, Hijlkema A, Streppel MT, Bautmans I, Weijs PJM, et al. Additional effects of exercise to hypocaloric diet on body weight, body composition, glycaemic control and

cardio-respiratory fitness in adults with overweight or obesity and type 2 diabetes: A systematic review and meta-analysis. Diabet Med 2023;40(7):e15096. doi: 10.1111/dme.15096.
15. Oja SS, Saransaari P. Taurine and the Brain. Adv Exp Med Biol 2022;1370:325-331. doi: 10.1007/978-3-030-93337-1_31.
16. Beutner F, Ritter C, Scholz M, Teren A, Holdt LM, Teupser D, et al. A metabolomic approach to identify the link between sports activity and atheroprotection. Eur J Prev Cardiol 2022;29(3):436-444. doi: 10.1093/eurjpc/zwaa122
17. **Ho KM, Lee A, Wu W, Chan MTV, Ling L, Lipman J, et al. Flattening the biological age curve by improving metabolic health: to taurine or not to taurine, that's the question. J Geriatr Cardiol 2023;20(11):813-823. doi: 10.26599/1671-5411.2023.11.004.**
18. Izquierdo JM. Taurine as a possible therapy for immunosenescence and inflammaging. Cell Mol Immunol 2024;21(1):3-5. doi: 10.1038/s41423-023-01062-5.
19. Duszka K. Versatile Triad Alliance: Bile Acid, Taurine and Microbiota. Cells 2022;11(15):2337. doi: 10.3390/cells11152337.
20. Christiansen CB, Trammell SAJ, Wewer Albrechtsen NJ, Schoonjans K, Albrechtsen R, Gillum MP, et al. Bile acids drive colonic secretion of glucagon-like-peptide 1 and peptide-YY in rodents. Am J Physiol Gastrointest Liver Physiol 2019;316(5):G574-584. doi: 10.1152/ajpgi.00010.2019.
21. Reilly SJ, O'Shea EM, Andersson U, O'Byrne J, Alexson SE, Hunt MC. A peroxisomal acyltransferase in mouse identifies a novel pathway for taurine conjugation of fatty acids. FASEB J 2007;21(1):99-107. doi: 10.1096/fj.06-6919com
22. Grevengoed TJ, Trammell SAJ, McKinney MK, Petersen N, Cardone RL, Svenningsen JS, et al. N-acyl taurines are endogenous lipid messengers that improve glucose homeostasis. Proc Natl Acad Sci U S A 2019;116(49):24770-24778. doi: 10.1073/pnas.1916288116.
23. Kim HW, Lee AJ, You S, Park T, Lee DH. Characterization of taurine as inhibitor of sodium glucose transporter. Adv Exp Med Biol 2006;583:137-145. doi: 10.1007/978-0-387-33504-9_14.
24. Tsuchiya Y, Kawamata K. Effects of taurine on plasma glucose concentration and active glucose transport in the small intestine. Anim Sci J 2017;88(11):1763-1767. doi: 10.1111/asj.12829.
25. Asnicar F, Berry SE, Valdes AM, Nguyen LH, Piccinno G, Drew DA, et al. Microbiome connections with host metabolism and habitual diet from 1,098 deeply phenotyped individuals. Nat Med 2021;27(2):321-332. doi: 10.1038/s41591-020-01183-8.
26. Collins SL, Stine JG, Bisanz JE, Okafor CD, Patterson AD. Bile acids and the gut microbiota: metabolic interactions and impacts on disease. Nat Rev Microbiol 2023;21(4):236-247. doi: 10.1038/s41579-022-00805-x.
27. Dobbins RL, Greenway FL, Chen L, Liu Y, Breed SL, Andrews SM, et al. Selective sodium-dependent glucose transporter 1 inhibitors block glucose absorption and impair glucose-dependent insulinotropic peptide release. Am J Physiol Gastrointest Liver Physiol 2015;308(11):G946-954. doi: 10.1152/ajpgi.00286.2014.
28. Li G, Tang T, Peng M, He H, Yin D. Direct reaction of taurine with malondialdehyde: evidence for taurine as a scavenger of reactive carbonyl species. Redox Rep 2010;15(6):268-274. doi: 10.1179/135100010X12826446921743.
29. Selvaraj N, Bobby Z, Sathiyapriya V. Effect of lipid peroxides and antioxidants on glycation of hemoglobin: an in vitro study on human erythrocytes. Clin Chim Acta 2006;366(1-2):190-195. doi: 10.1016/j.cca.2005.10.002.
30. Esmaeili F, Maleki V, Kheirouri S, Alizadeh M. The Effects of Taurine Supplementation on Metabolic Profiles, Pentosidine, Soluble Receptor of Advanced Glycation End Products and Methylglyoxal in Adults With Type 2 Diabetes: A Randomized, Double-Blind, Placebo-Controlled Trial. Can J Diabetes 2021;45(1):39-46. doi: 10.1016/j.jcjd.2020.05.004.

31. EFSA Panel on Additives and Products or Substances used in Animal Feed (FEEDAP). Scientific Opinion on the safety and efficacy of taurine as a feed additive for all animal species. The EFSA Journal 2012;10(6):2736. doi:10.2903/j.efsa.2012.2736.
32. Levine ME, Lu AT, Quach A, Chen BH, Assimes TL, Bandinelli S, et al. An epigenetic biomarker of aging for lifespan and healthspan. Aging (Albany NY) 2018;10(4):573-591. doi: 10.18632/aging.101414.
33. Zhou H, Lee JJ, Yuan Y. BOP2: Bayesian optimal design for phase II clinical trials with simple and complex endpoints. Stat Med 2017;36(21):3302-3314. Doi:10.1002/sim.7338.
34. Ho KM, Lee A. Using Bayesian hypothesis-testing to reanalyse randomized controlled trials: does it always tell the truth the whole truth and nothing but the truth? Indian Journal of Critical Care Medicine 2024 (in press).
35. Suliman ME, Bárány P, Filho JC, Lindholm B, Bergström J. Accumulation of taurine in patients with renal failure. Nephrol Dial Transplant 2002;17(3):528-529. doi: 10.1093/ndt/17.3.528.
36. Roşca AE, Vlădăreanu AM, Mirica R, Anghel-Timaru CM, Mititelu A, Popescu BO, et al. Taurine and Its Derivatives: Analysis of the Inhibitory Effect on Platelet Function and Their Antithrombotic Potential. J Clin Med 2022;11(3):666. doi: 10.3390/jcm11030666.
37. Schaffer SW, Jong CJ, Ramila KC, Ito T, Kramer J. Differences Between Physiological and Pharmacological Actions of Taurine. Adv Exp Med Biol 2022;1370:311-321. doi: 10.1007/978-3-030-93337-1_30.
38. Cleland C, Ferguson S, Ellis G, Hunter RF. Validity of the International Physical Activity Questionnaire (IPAQ) for assessing moderate-to-vigorous physical activity and sedentary behaviour of older adults in the United Kingdom. BMC Med Res Methodol 2018;18(1):176. doi: 10.1186/s12874-018-0642-3.
39. Du X, Liao J, Ye Q, Wu H. Multidimensional Internet Use, Social Participation, and Depression Among Middle-Aged and Elderly Chinese Individuals: Nationwide Cross-Sectional Study. J Med Internet Res 2023;25:e44514. doi: 10.2196/44514.
40. Atzeni IM, van de Zande SC, Westra J, Zwerver J, Smit AJ, Mulder DJ. The AGE Reader: A non-invasive method to assess long-term tissue damage. Methods 2022;203:533-541. doi: 10.1016/j.ymeth.2021.02.016.
41. U.S. Food and Drug Administration. FDA Food Substances Database: Lactose [Internet]. Silver Spring (MD): FDA; [cited 2025 Sep 18]. Available from: https://www.hfpappexternal.fda.gov/scripts/fdcc/index.cfm?set=FoodSubstances&id=LACTOSE
42. Dalal SR, Chang EB. Disorders of epithelial transport, metabolism, and digestion in the small intestine. In: Podolsky DK, Camilleri M, Fitz JG, Kalloo AN, Shanahan F, Wang TC, editors. *Yamada's Textbook of Gastroenterology*. 6th ed. West Sussex: John Wiley & Sons; 2016. p. 1276–93.

CONSENT TO PARTICIPATE IN A RESEARCH STUDY

Department of Anaesthesia and Intensive Care
The Chinese University of Hong Kong

Title of study

Taurine or Placebo for Healthcare Workers: the TOP Healthcare workers Bayesian Optimized Phase II randomized-controlled trial

Background

This study is led by Professor Kwok Ming HO.

Taurine is a conditionally essential amino acid that exists in high concentrations in multiple organs such as the brain, heart, retina, and skeletal muscles. In nonhuman studies, taurine is shown to be effective in improving cellular and organ function including their metabolic health such as blood sugar level, bone density and mental health. Current nutrition-related studies largely focus on macronutrients, with insufficient human data to support the health benefits brought by taurine. Taurine is contained in many seafood items and in some dark meat, but its intake can vary substantially between individuals.

Having a healthy healthcare workforce is one of the ways to reduce the healthcare burden and achieve a sustainable healthcare system. As a healthcare-related professional, we believe you would be interested in maintaining your health and hence we cordially invite you to participate in this study to help evaluate the health benefits of taking taurine orally for 6 months.

The objectives of this study are to evaluate whether taurine can

1. improve your blood glucose metabolism profile and
2. improve your blood low-density-lipoprotein (LDL) and triglyceride levels, biological age – as measured by the PhenoAge model – and accumulation of advanced glycation end-products (AGEs); and
3. whether the potential benefits described above will be dependent on one's chronological age and underlying diabetic status

Procedures

You will be randomly assigned to either the **Placebo** group or the **Intervention** group by computer-generated sequence.

Depending on your group allocation, you will be given identical-looking placebo or taurine study capsules and take 3 capsules orally in one sitting per day for 6 months. The placebo is high-purity, spray-dried, National Formulary standard, lactose monohydrate that is a kind of purified milk sugar and free of milk protein. It is considered as a safe food substance according to the U.S. Food and

Drug Administration. This taurine dose (1000mg/capsule x 3) is much higher than the standard daily dietary intake, yet well within the safe recommended dose. The capsules are best to be taken in the morning, but you may take them at other time during the day. You may continue your normal diet. You may also continue your usual medication, if any, but one hour apart from taking the study capsules.
After agreeing to participate in this study, you may also be invited to participate in our sub-study about gut microbiota diversity in relation to changes in plasma taurine-conjugated bile acid and methylglyoxal levels.
Throughout the study, you will be required to visit hospitals of New Territories East Cluster twice for assessments including:

- **blood tests** for HbA1c, LDL and triglyceride levels, biological age, body weight,
- **2 sets of questionnaires** (International Physical Activity Questionnaire (IPAQ) and Center for Epidemiologic Studies Depression Scale (CES-D)),
- **blood pressure**
- **dietary habit (such as no seafood, vegan etc)**,
- **AGEs index** (measured non-invasive using the skin autofluorescence technology).

The first assessment will be done (at baseline) before you start taking the study capsules. The second assessment will be done at the 6-month follow-up.
On the day of the blood tests at the 6-month follow-up, you will need to take the study capsules in the morning between 1 and 3 hours before blood sample collection.
If you are invited to participate in our sub-study, stool samples will be collected before the study begins and at the 6-month follow-up.

Specimen collection and handling
All investigations and specimens will be done and collected in Prince of Wales Hospital from healthcare workers in hospitals of the New Territories East Cluster including Prince of Wales Hospital, North District Hospital, and Alice Ho Miu Ling Nethersole Hospital. Blood tests for taurine, and methylglyoxal levels will be analysed by the ELISA assays.
Stool samples will be aliquoted into cell culture cryogenic tubes and immediately stored at -80°C for metagenomic sequencing locally.
Specimen will be stored up to 3 years as the samples will only be shipped (in dry ice) and analyzed in batches or as a whole when enrollment and follow-up have been completed.

Risks and benefits
You may experience the positive effects brought by taurine such as the improvement in blood glucose level, exercise capacity, and a lower blood pressure, yet results may vary from person to person as lacking scientific proofs. Although side effects of taurine are rare and have not been

considered as a concern for taking taurine according to the literature and previous human trials, it is important for you to report to us any adverse effects you experience once you start taking the study capsules, these may include gastrointestinal upset, allergic skin rashes, dizziness that caused by spontaneous low blood sugar or reduction in blood pressure not due to your other medications such as oral hypoglycaemic agents (if you are diabetic), unexpected bleeding from any sites after the commencement of study capsules, and any new symptoms you believe are likely related to the study medications.

Adverse effects that you may experience will be monitored by a two-member independent data safety monitoring committee (DSMC). The study will be ceased if the pre-plan safety margin is reached. Your participation is purely voluntary. Should you experience adverse effects or experience symptoms that believed to be related to the study capsules, and unwilling to resume the study capsules, you have the right to leave the trial without consequences. At the end of the study, we would like you to return the unused capsules to us so that we estimate the total amount of taurine you have taken.

Other than lactose monohydrate, the study placebo contains no other substance. There is no risk from taking the placebo as it is free from milk protein. Existing research also shows that people with lactose intolerance are able to intake up to 12 grams of lactose with only mild symptoms or even no symptoms.

All assessments and the taurine/placebo capsules this study involves will be provided to you for free. However, joining this study will not provide you with any monetary benefit. Your participation will be completely voluntary. You may request to withdraw from the study at any stage of the study without any consequences. The blood taking procedure for this study is similar to routine blood taking. Potential risks may include temporary discomfort, bruising, swelling or the possibility of bleeding at the puncture site. Aseptic technique will be applied to reduce the risk of infection. The frequency of these is not expected to be any different for participants in this study as compared to routine blood taking for other clinical purposes.

Our team will ensure minimization and prompt identification of these potential risks at our best afford.

Ethics Approval

This study has been approved by the Joint Chinese University of Hong Kong – New Territories East Cluster Clinical Research Ethics Committee (NTEC-CUHK Cluster REC/IRB: 2024.337T) (phone: 3505-3935).

Confidentiality

All information collected in this study will be used for research purposes only and will be considered confidential. All personal information will be handled and kept confidential in accordance with the Personal Data (Privacy) Ordinance (Cap. 486) of the Hong Kong Special

Administrative Region. Only authorized parties such as NTEC-CUHK Cluster REC/IRB can access your records regarding ethics review purposes. All data will be deleted 7 years after publication of research papers.

Concerns and inquiries

If you have further inquiries, you can contact Ms Grace HOU (research nurse) or Professor Kwok Ming HO (principal investigator) at 35052735. If you have further inquiries about your right as a study participant, please do not hesitate to contact our Joint Chinese University of Hong – New Territories Easter Cluster Clinical Research Ethics Committee at 35053935.

CONSENT TO PARTICIPATE IN A RESEARCH STUDY

Department of Anaesthesia and Intensive Care
The Chinese University of Hong Kong

Patient demographic information

Title of study

Taurine or Placebo for Healthcare Workers: the TOP Healthcare workers Bayesian Optimized Phase II randomized-controlled trial

Main study consent

I agree to participate in this study. I have read the information provided and understand the explanation that has been provided to me and understand that I have the right to cease to participate in this study at any time.

……………………………………	……………………………………
Name of participant	Name of research assistant/investigator/nurse
……………………………………	……………………………………
Signature of participant	Signature of research assistant/investigator/nurse
……………………………………	……………………………………
Date	Date

Gut microbiome sub-study study consent

I agree to participate in the sub-study. I have read the information provided and understand the explanation provided to me.

……………………………………	……………………………………
Name of participant	Name of research assistant/investigator/nurse
……………………………………	……………………………………
Signature of participant	Signature of research assistant/investigator/nurse
……………………………………	……………………………………
Date	Date

This study will require you to have blood tests:
(1) before the start of taking the study capsules
(2) at the 6-month follow-up.

If you are invited and agree to participate in the sub-study, the study will collect stool samples from you twice at the same schedule as blood tests.

Would you like to receive the following information after the whole trial is completed?

1. The results of the main study

☐ Yes ☐ No

2. All blood and study sample results

☐ Yes ☐ No

3. Your group allocation

☐ Yes ☐ No

參與研究同意書

威爾斯親王醫院麻醉及深切治療部

研究項目

醫護人員接受牛磺酸或安慰劑貝葉斯優化的 II 期隨機對照試驗

背景資料

本研究項目是由 何國明教授帶領。

牛磺酸是一種半必需胺基酸，大量存在於大腦、心臟、視網膜和骨骼肌等多個器官。 在非人類的動物研究中發現牛磺酸可以有效地改善細胞和器官功能，包括代謝健康，如血糖水平、骨質密度和心理健康。 目前相關的營養研究主要集中在巨量營養素上，而牛磺酸對人類的健康益處數據並不足夠。 許多海鮮食品和一些深色肉類都含有牛磺酸，但其攝取量卻因人而異。

擁有一支健康的醫療團隊是減輕醫療負擔和實現永續醫療保健系統的方法之一。 我們相信您作為醫療專業人士會對保持健康感到興趣，因此我們誠摯邀請您參與這項研究，以幫助我們評估口服 6 個月的牛磺酸對健康的益處。

本研究的目的是評估牛磺酸是否可以

1. 改善您的血糖代謝狀況
2. 改善血液低密度膽固醇 (LDL) 和三酸甘油酯水平、生物年齡（透過 PhenoAge 模型測量）、以及糖化終產物 (AGE) 的累積
3. 以上提及的潛在益處取決於一個人的實際年齡和患有糖尿病的狀況

程序

您將按照電腦產生的序列被隨機分配到安慰劑組或介入組。

根據隨機分配結果，您將獲得外觀相同的安慰劑或牛磺酸研究膠囊，每天一次口服 3 粒膠囊，持續 6 個月。安慰劑為乳糖一水合物，是一種純化的乳糖，不含牛奶蛋白。根據美國食品藥物管理局（FDA）的資料，它被視為一種安全的食品物質。牛磺酸的劑量（1000 毫克/膠囊 x 3）遠高於每日的標準攝取量，但完全在安全建議劑量內。膠囊最好在早上服用，但您也可以在白天的其他時間服用。繼續正常飲食，如果有常用藥物也可以繼續服用，但需要與研究膠囊相隔一小時。

同意參加本研究，我們也希望邀請您參加腸道微生物群多樣性與血漿牛磺酸結合膽汁酸和甲基乙二醛水平變化相關的次研究。

在整個研究過程中，你需要前往新界東聯網的醫院兩次進行評估，包括：

- 血液檢測 : 糖化血色素(HbA1c)、低密度膽固醇 (LDL) 和三酸甘油酯水平，生物年齡和體重

- 2 套問卷（國際體育活動問卷（IPAQ）和流行病學研究中心憂鬱量表（CES-D））
- 血壓
- 飲食習慣（例如不吃海鮮，全素等）
- AGEs 指數（使用非侵入性皮膚自發螢光技術測量）

在您開始服用研究膠囊之前，會進行第一次評估作為基準線。第二次評估將在 第 6 個月跟進時進行。
在 第 6 個月跟進血液檢測的當天，您需要在早上採集血液樣本前 1 至 3 小時服用研究膠囊。
如果您有參加我們的次研究，我們將在研究開始前和第 6 個月跟進時收集糞便樣本。

樣本採集與處理
所有檢查及樣本收集都會在威爾斯親王醫院進行。所有樣本均來自新界東聯網醫院包括威爾斯親王醫院，北區醫院，及雅麗氏何妙齡那打素醫院之各級醫護同事。牛磺酸、 和甲基乙二醛水平的血液檢測將以酶聯免疫吸附測定方法進行分析。
糞便樣本將等分到細胞培養低溫管中，並立即儲存到攝氏 -80 度，在本地進行總體基因組定序分析。
樣本將會於參與者完成登記及 6 個月的跟進完成後分批或整批運輸（在乾冰中）及進行分析，所有樣本將會被儲存最多 3 年。

風險和益處
牛磺酸可能產生的正面作用，例如改善血糖水平、提高運動能力和降低血壓。因缺乏科學實證，效果可能因人而異。根據文獻和之前的人體試驗，牛磺酸的副作用很少見，而服用牛磺酸並不會造成問題，但您在開始服用研究膠囊後，如果有任何的副作用包括胃腸道不適、過敏性皮疹、自發性低血糖或低血壓 (並非由於您患有糖尿病服用降血糖藥或其他藥物引起)而引致的頭暈、開始服用研究膠囊後任何部位異常出血，或者您認為任何新症狀可能與研究藥物有關，我們都視為非常重要的資訊，請向我們報告。
您可能遇到的副作用將由兩名成員組成的獨立資料安全監測委員會 (DSMC) 進行監測。如果超出預先計劃的安全限度，研究將停止。您的參與純屬自願。如果您出現不良反應或出現被認為與研究膠囊有關的症狀，並且不願意再服用研究膠囊，您有權退出試驗而無需承擔任何後果。在研究結束時，我們希望您將未使用的膠囊歸還給我們，以便我們可以估算您服用的牛磺酸總量。
除了乳糖一水合物外，該研究的安慰劑不含其他物質。由於其不含牛奶蛋白，因此服用安慰劑是沒有風險的。現有研究亦顯示，乳糖不耐症患者即使攝取多達 12 克乳糖，也僅會出現輕微症狀，甚至可能完全沒有症狀。
本研究涉及的所有測試和評估以及牛磺酸補充劑/安慰劑膠囊將免費提供給你。然而，參加這項研究不會為您帶來任何金錢上的好處。您的參與將完全是自願性質。您可以在研究的任

何階段請求退出而不會產生任何後果。
本研究的抽血程序與常規的抽血程序大致相同。抽血可能會產生之生理影響，如短暫的不適，瘀青，腫脹或抽血部位流血。無菌技術會應用於抽血過程以減低感染發生機會。抽血的潛在風險發生的機會並不會因參與此研究二有所不同。
我們的研究團隊將會盡力及時識別並減低這些潛在風險並作出適切的處理以確保參與者的安全。

研究倫理批准
本研究已獲得香港中文大學-新界東聯網聯合臨床研究倫理委員會批准（NTEC-CUHK Cluster REC/IRB：2024.3374T 電話：3505-3935）。

保密
本研究收集的所有資訊會被視為機密和僅用於研究。所有關於你的個人資料，我們會依照香港特別行政區《個人資料（隱私）條例》(Cap.486) 保密處理。只有香港中文大學 - 新界東醫院聯網臨床研究倫理聯席委員會授權而且出於道德審查目的方能存取您的記錄。所有數據將在研究論文發表 7 年後刪除。

疑問和查詢
如果您有進一步的疑問，您可以聯絡侯碧怡女士（研究護士）或何國明教授（首席研究員），電話：35052735。如果您對您作為研究參與者的權利有進一步的疑問，請隨時聯絡香港中文大學 - 新界東醫院聯網臨床研究倫理聯席委員會，電話：35053935。

參與研究同意書

威爾斯親王醫院麻醉及深切治療部

Patient 個人資料

研究項目

醫護人員接受牛磺酸或安慰劑貝葉斯優化的 II 期隨機對照試驗

主研究同意書

我同意參加這項研究。我已閱讀所提供的資訊並理解向我提供的解釋，我知道我有權隨時停止參與這項研究。

……………………………………	……………………………………
參與者姓名	研究助理員/調查員/護士姓名
……………………………………	……………………………………
參與者簽署	研究助理員/調查員/護士簽署
……………………………………	……………………………………
日期	日期

腸道微生物組次研究同意書

我同意參加這項子研究。我已閱讀所提供的資訊並理解所提供的解釋。

……………………………………	……………………………………
參與者姓名	研究助理員/調查員/護士姓名
……………………………………	……………………………………
參與者簽署	研究助理員/調查員/護士簽署
……………………………………	……………………………………
日期	日期

這項研究將要抽取您的血液檢測：
(1) 開始服用研究膠囊前
(2) 第 6 個月跟進時

如果您受邀並同意參加這次研究，研究將按照與血液檢測相同的時間表從您那裡收集兩次糞便樣本。

您希望在整個試用結束後收到以下資訊嗎？

1. 主要研究結果

   ☐ 要　☐ 不要

2. 所有血液和研究樣本結果

   ☐ 要　☐ 不要

3. 你被分配的組別

   ☐ 要　☐ 不要
